# Spatial models for the rational allocation of routinely distributed bed nets to public health facilities in Western Kenya

**DOI:** 10.1186/s12936-017-2009-3

**Published:** 2017-09-12

**Authors:** Peter M. Macharia, Patroba A. Odera, Robert W. Snow, Abdisalan M. Noor

**Affiliations:** 1 Department of Geomatic Engineering and Geospatial Information Systems, Jomo Kenyatta University of Agriculture and Technology, Nairobi, Kenya; 2 Kenya Medical Research Institute/Wellcome Trust Research Programme, P.O. Box 43640-00100, Nairobi, Kenya; 3 Division of Geomatics, School of Architecture, Planning and Geomatics, University of Cape Town, Cape Town, South Africa; 4 Centre for Tropical Medicine and Global Health, Nuffield Department of Clinical Medicine, University of Oxford, Oxford, UK

**Keywords:** LLINs allocation, ANC utilization, Spatial modelling, Equity

## Abstract

**Background:**

In high to moderate malaria transmission areas of Kenya, long-lasting insecticidal nets (LLINs) are provided free of charge to pregnant women and infants during routine antenatal care (ANC) and immunization respectively. Quantities of LLINs distributed to clinics are quantified based on a combination of monthly consumption data and population size of target counties. However, this approach has been shown to lead to stock-outs in targeted clinics. In this study, a novel LLINs need quantification approach for clinics in the routine distribution system was developed. The estimated need was then compared to the actual allocation to identify potential areas of LLIN over- or under-allocation in the high malaria transmission areas of Western Kenya.

**Methods:**

A geocoded database of public health facilities was developed and linked to monthly LLIN allocation. A network analysis approach was implemented using the location of all public clinics and topographic layers to model travel time. Estimated travel time, socio-economic and ANC attendance data were used to model clinic catchment areas and the probability of ANC service use within these catchments. These were used to define the number of catchment population who were likely to use these clinics for the year 2015 equivalent to LLIN need. Actual LLIN allocation was compared with the estimated need. Clinics were then classified based on whether allocation matched with the need, and if not, whether they were over or under-allocated.

**Results:**

888 (70%) public health facilities were allocated 591,880 LLINs in 2015. Approximately 682,377 (93%) pregnant women and infants were likely to have attended an LLIN clinic. 36% of the clinics had more LLIN than was needed (over-allocated) while 43% had received less (under-allocated). Increasing efficiency of allocation by diverting over supply of LLIN to clinics with less stock and fully covering 43 clinics that did not receive nets in 2015 would allow for complete matching of need with distribution.

**Conclusion:**

The proposed spatial modelling framework presents a rationale for equitable allocation of routine LLINs and could be used for quantification of other maternal and child health commodities applicable in different settings. Western Kenya region received adequate LLINs for routine distribution in line with government of Kenya targets, however, the model shows important inefficiencies in the allocation of the LLINs at clinic level.

## Background

Long-lasting insecticidal nets (LLINs) are distributed as the main tool of vector control for malaria prevention in Africa, through mass campaigns and routine distribution systems [1]. Mass campaigns, target population at community level in a single time-limited operation every 3 years. Routine systems deliver LLINs through public health sector all year round to sustain coverage in the interval between mass campaigns to pregnant women and infants who are the most vulnerable. The World Health Organization (WHO) recommends both channels for countries in order to achieve and maintain universal coverage [1, 2].

At the launch of Kenya’s national malaria strategy in 2001, the aim was to achieve 60% insecticide-treated nets (ITNs) coverage of at risk children and pregnant women by 2006 [3]. Prior to this, access to ITNs was limited to the private sector, a few research projects and non-governmental organizations. Different channels of ITNs delivery were explored including commercial retail sector ITNs and subsidized ITNs through public health sector. These approaches did not succeed in reaching the rural poor and achieving maximum coverage of the population at risk [4]. In 2004, free routine LLIN distribution in antenatal care clinics (ANC) began and in 2006 the first free mass-campaign was conducted. Subsequent free mass campaigns were conducted in 2011, 2012, 2014 and 2015 in malaria endemic areas of Kenya [5].

Routine distribution of LLINs in Kenya are targeted to high and moderate malaria transmission areas, where pregnant women are provided with a free LLIN during their first ANC visit so that the mother and the unborn baby are protected at the earliest chance. Infants are provided with a free LLIN during expanded programme on immunization (EPI) [6, 7]. There is no specific WHO recommendation as to when LLINs should be distributed during EPI. However, a range of time-points is used in practice, from birth (tuberculosis vaccine) to 9 months (measles vaccine). Diphtheria-tetanus-pertussis-1 vaccination at 6 weeks is the most common distribution point [7, 8]. ANC clinics that distribute LLINs have increased rapidly from 1000 nationally in 2004 to over 4000 in 2015, with a total 23.3 million LLINs distributed [5].

Pregnant women receive a package of interventions during ANC visits which play an important role in ensuring a healthy mother and baby during pregnancy and after delivery [6, 9]. Healthcare utilization for ANC services is associated with intrapersonal, institutional, health systems and social demographic factors [10, 11]. Similarly, the ownership and use of LLINs during pregnancy is associated with household wealth, distance to health facility, residence, parity, marital status, and education among others [12]. It is key, therefore, to include these spatial variables to account for the uneven healthcare utilization rates within facility catchment areas within a spatial modelling framework [13, 14].

The quantity of LLINs distributed to clinics is quantified at the central level using average monthly LLINs consumption data and the overall size of the population in targeted counties. However, this approach has resulted in frequent stock-outs [7, 15] with subsequent low population coverage of LLIN among pregnant women and infants [8, 15, 16]. In this study, a spatial modelling framework was proposed and used to quantify LLINs need by ANC clinic to allow more efficient targeting. A combination of modelled spatial access to clinics, household survey data on utilization and high resolution population grids were used to quantify the population of pregnant women and infants in 2015 in need of LLINs. The estimated need was compared to the actual allocation in 2015 and clinics with fewer (*under*-*allocation*), more (*over*-*allocation*) and enough (*matched*-*allocation*) LLIN relative to the estimated need identified. A fourth category is those facilities with zero distribution (*non*-*allocation*). Approaches to minimize targeting inefficiencies were then proposed.

## Methods

### Data

**Fig. 1:**
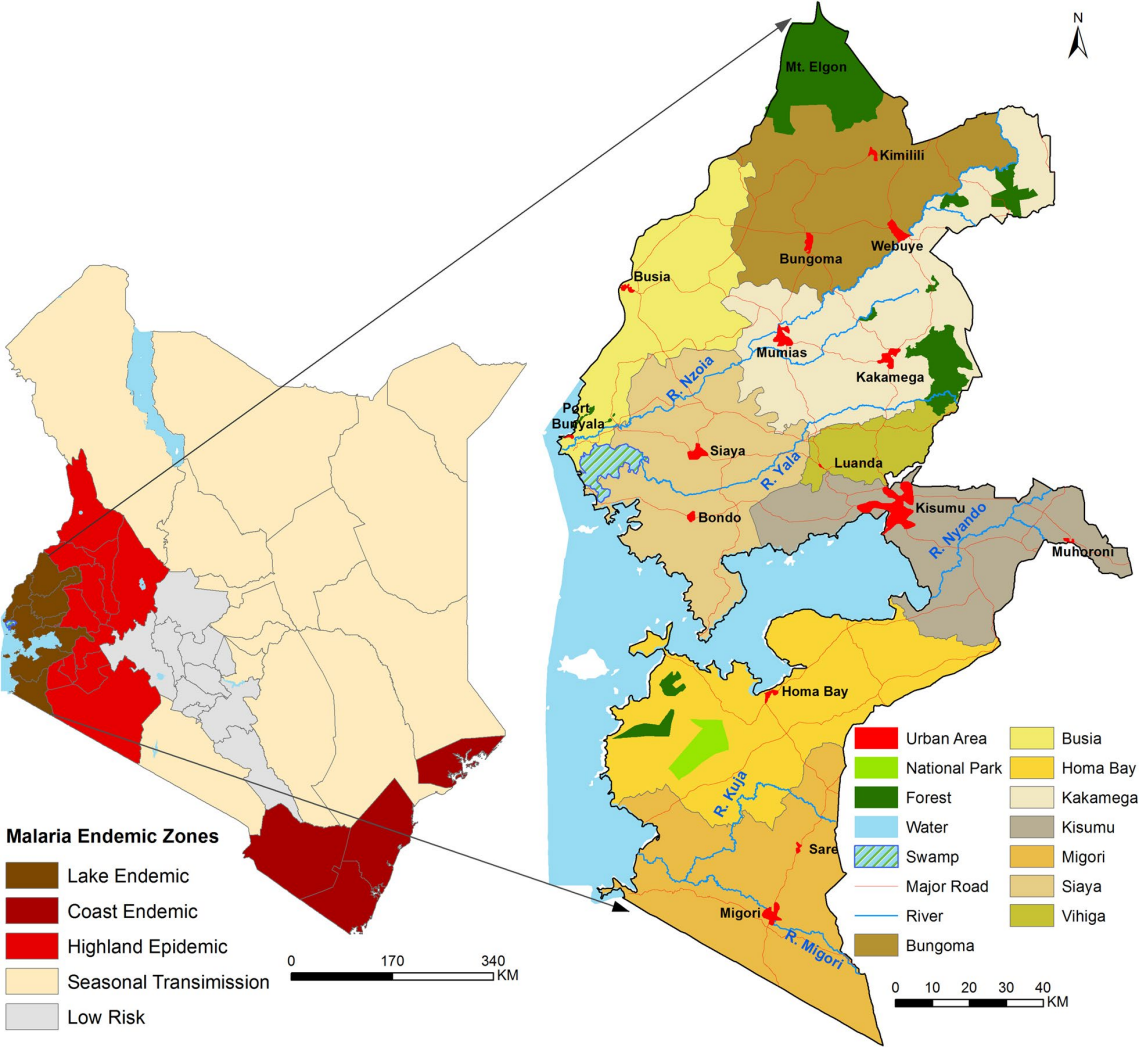
Maps of; Kenya five malaria endemicity zones (**a**) and eight study counties in Lake Endemic zone with high, stable malaria transmission throughout the year (**b**)

Kenya is stratified into five malaria zones to address varied risks of malaria (Fig. 1a). They include Lake endemic (high, stable perennial transmission areas), Coast endemic (moderate, seasonal transmission areas), seasonal low transmission (acutely seasonal, low transmission areas), Highland epidemic (unstable, variable transmission areas) and Low risk (malaria free or extremely low transmission) [17, 18]. The analysis was restricted to the Lake endemic zone (Fig. 1b), because of its high, stable transmission throughout the year and presence of routine LLIN distribution to pregnant women and infants since 2004. In this zone, *Plasmodium falciparum* prevalence has historically been greater than 20%. The zone comprises of eight counties; Busia, Siaya, Bungoma, Homa Bay, Kakamega, Kisumu, Migori, and Vihiga (Fig. 1b) [5].

Previously mapped health facilities [19] were updated using the Ministry of Health [20] and the District Health Information System version 2 (DHIS 2) lists [21]. Lists were compared to eliminate duplicates. Health facilities managed by the government, faith-based and non-governmental organizations were selected as public health facilities. Health facilities that did not have coordinates were geo-located using Google Earth [22]. Monthly LLIN distribution data by clinic were obtained from Population Services Kenya and linked to the geocoded database.

Livebirths and pregnancies distribution maps for 2015 at 100 × 100 m spatial resolution were downloaded from WorldPop data portal [23]. These maps were constructed using dasymetric spatial modelling techniques that redistributed the Kenya’s national census (2009) population counts within 6603 sub-locations [24, 25]. Population was shifted away from areas unlikely to be inhabited using weights assigned to each land cover with national estimates projected to match 2015 UN national estimates [26]. Population surfaces were grouped by sex and 5 year-age groups and adjusted using age-specific fertility rates for females of childbearing age [27]. The resultant live births surface was adjusted to match the Guttmacher Institute estimates [28] and converted to pregnancies by adjusting national totals to match national estimates [29]. The livebirths and pregnancy surfaces was aggregated to 300 m spatial resolution while all the other surfaces were resampled to the same resolution in ArcGIS version 10.1 (ESRI Inc., Redlands, CA, USA) for ease of computation and compatibility with the travel time surface which was modelled at 300 m spatial resolution.

Socio-economic, demographic and ANC attendance data for pregnant women were obtained from the 2014 Kenya Demographic and Health Survey (KDHS). This survey employed a two-stage sampling design on a national sampling frame of 5360 clusters. In the first stage, 1612 clusters (995 in urban areas and 617 in rural areas) were selected with equal probability while in the second stage, 40,300 households were selected from the household listing of the 1612 clusters. More than 19% (2776) of all women in the reproductive age (15–49 years) who had a live birth 5 years preceding the survey in Kenya, were residents of 277 clusters (181 in rural and 96 in the urban areas) in the 8 study counties, all geo-located [30].

In order to protect and maintain the confidentiality of the respondents in the KDHS, Global Positioning System coordinates (latitude and longitude) were randomly displaced up to 2 km for urban clusters and 5 km for rural clusters with 1% of the rural clusters displaced up to 10 km [31]. In this study, the scrambling effect was minimized by drawing 2 km (urban) and 5 km (rural) Euclidean buffers around the cluster points, which were then shifted to their most probable location within the buffer [32–34]. This was based on a point’s elevation (not scrambled) provided along with the coordinates and a digital elevation model (DEM). To supplement the elevation information, Google Earth [22] and population distribution surfaces [24] were used to relocate the point in populated or urban location within the buffer.

Road network data from OpenStreetMaps (OSM) [35] and Google Map Maker (GMM) [36] was updated via Google Earth [22]. Duplicates and short sections of roads disconnected from the main network were removed in the resultant road network. Roads were then classified based on the inherent classification on OSM, GMM, Google Earth, the Kenya Roads Act [37] and Bill [38]. The Bill and the Act outlines the criteria for classifying public roads in Kenya including primary, secondary, county and rural roads (Table 1). The road network was converted into a raster surface. Land cover at 30 m spatial resolution was downloaded from China’s global mapping project [39] generated from Landsat and Environmental Disaster Alleviation Satellites imagery. DEM at 30 m spatial resolution by Shuttle Radar Topographic Mission was obtained from Earth Explorer portal [40].

**Table 1:** Description of data types, mode of travel (motorized, cycling and walking) and speeds used in the modelling travel time to public health facilities distributing LLINs in Western region of Kenya

Data (mode of transport)	Road class	Description	Speed (km/hr)
Primary road (vehicular)	A	Strategic corridors connecting international boundaries at specific immigration and entry points	50
	B	Link national trading and economic hubs, county headquarters, important national centers and connects to class A road	50
Secondary road (vehicular)	C	Link county and regional headquarters to each other and to roads of class A or B	30
	D	Link constituency headquarters, town centers and other municipal centers to each other and to higher-class roads	30
County road (bicycling)	E	Major feeder roads linking important constituency centers They carry local traffic and link constituency centers	11
	G	Carry’s farm produce/inputs to and from the markets	11
	L	Collect traffic from the local roads to the arterial roads	11
Rural roads (walking)	R	Roads accessing rural areas	5
	S	Roads accessing sugarcane growing areas	5
	T	Roads accessing tea growing areas	5
	U	Unclassified rural roads	5
Wetland (walking)	Include inland marsh lake, floodplain wetland, forest/shrub wetland, peat bogs, mangrove and salt marsh etc		1
Shrub land (walking)	Covered with shrubs (>30%) including deciduous and evergreen shrubs, and desert steppe (>10%)		4
Grassland (walking)	Lands covered by natural grass cover over 10%		3
Cultivated land (walking)	Lands used for agriculture, horticulture gardens, including paddy fields, irrigated and dry farmland, vegetation and fruit gardens		5
Artificial surfaces (walking)	Lands modified by human activities, including all kinds of habitation, industrial and mining area, transportation facilities and interior urban green zones		5

The travel speeds are based on previous comparable studies

### Estimating LLINs need per clinic

**Fig. 2:**
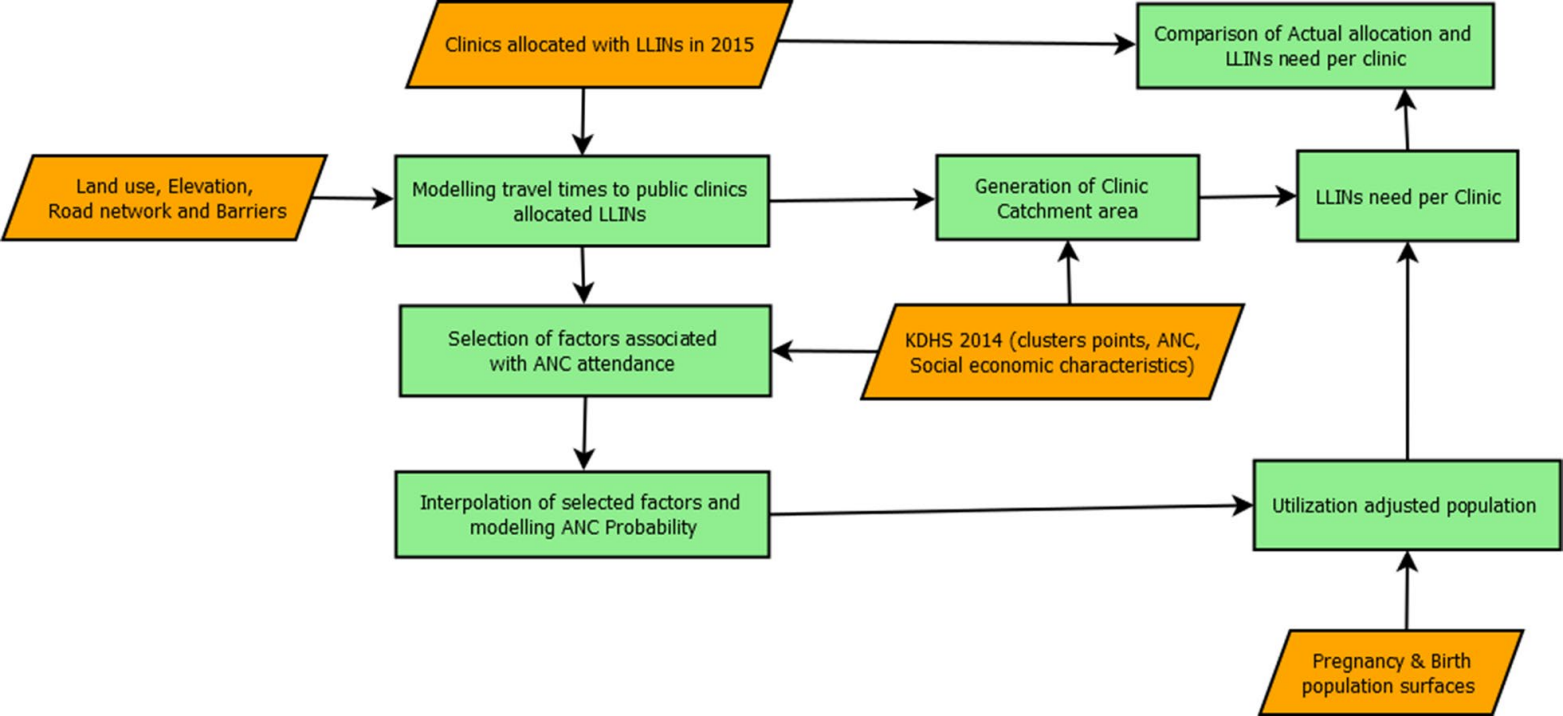
Analytical process used to quantify LLIN need and mis-allocation at each clinic in Western Kenya. The datasets are shown in *orange parallelograms* while processes are shown in *green rectangles*

A flowchart showing the analytical process is presented in Fig. 2. Travel time and catchment areas for clinics were modeled. Within each catchment, the total population and proportion of pregnant women and infants in 2015 were estimated. Probability surface of ANC utilization was then modeled and used to adjust catchment population per ANC clinic to define those who are most likely to use it. The catchment population of pregnant women and infants for 2015 was then extracted per clinic and these were used as equivalent to the estimated LLIN need per clinic. This was then compared to the 2015 actual LLIN allocation.

To implement the travel time analysis, different speeds were assigned on the road network depending on the likelihood of using motorized, bicycling and walking travel modes (Table 1). Speeds assigned to road types and land cover classes were adopted from comparable studies [13, 41–43]. Slope derived from DEM was used to adjust walking [44] and bicycling speeds [45, 46]. The roads and land use grids were then combined into a single travel time raster grid by adding up the time needed to cross contiguous cells to the nearest facility via the shortest path in ArcView (version 3.2) using AccessMod (version 3.0) at 300 m spatial resolution for computation ease [13, 47].

For the analysis of the probability of utilization of ANC clinics, travel times were extracted for each KDHS 2014 cluster location. A distance decay curve was fitted to predict the probability of attendance with travel time [48] using a three parameter logistic regression [13]. A *cut*-*off* time within which most pregnant women accessed a clinic was determined through the curve inflection point. The cut-off time and the travel time grid were used to define facility catchment areas [49] using *Path Distance* tool in ArcGIS.

To select factors associated with ANC attendance among those extracted from KDHS 2014 (wealth quintiles, maternal education, parity, maternal age, residence, marital status) and the modelled time, a hierarchical mixed effects logistic regression model (Eq. 1) was implemented using “*lme4*” package [32, 33, 50]. The parsimonious model was defined by the model with the lowest Akaike Information Criterion value [51].

1 $$\text{logit} (P) = \alpha + \beta X + Z\gamma$$

where **P**- is the probability of ANC attendance, **α**-the intercept; **β-** the vector of unknown regression coefficients for the fixed effects; **X**- a matrix of known covariates (wealth, education, parity, age, residence, marital status and travel time. **Z**- a matrix of random effect (clusters nested within counties); **γ**- a vector of variance. A binary outcome was defined; 1 (1+ ANC visits) and 0 (0 ANC visit).

Spatial structure of the selected variables was assessed using variogram while interpolation was implemented using ordinary kriging in ‘*geoR’* package, with a 10% hold out for validation [52]. The travel time and the interpolated surfaces were used to define an ANC attendance probability surface (Eq. 2) in R software.

2 $$\text{P} (Y) = \left( {\frac{N}{{(1 - e^{ - (\alpha + \beta X)} )}}} \right)$$

(**β**)- are the coefficients of the matrix of known covariates (**X**) and the intercept **(α)** from Eq. 1. **N** is a limiting factor on y axis. It defines the probability of attendance at zero minutes and in presence of ideal circumstances for other influencing variables (e.g. higher wealth quantile and higher maternal education). However, other variables including but not limited to the unmeasurable and unmeasured variables limit the probability to less than 1. All the other symbols have similar meaning as those in Eq. 1.

The gridded maps of pregnant women and live births in 2015 were summed together and the population per clinic catchment extracted. In addition, the summed population was multiplied with the ANC probability surface to get those likely to have used a clinic. Those likely to have used a clinic were extracted per each clinic catchment using Zonal statistics tool in ArcGIS.

### LLIN allocation efficiency at each clinic

The difference between total population and those likely to have used a clinic per catchment area on average was a 100 people (both pregnant women and infants). Consequently, *over*-*allocation* was defined as >100 LLINs allocation higher than the need, under-*allocation* as >100 LLINs allocation lower than the need and matched-allocation as ±100 LLINs relative to the need. *Non*-*allocated* areas were defined as those with clinics where no LLINs were distributed in 2015. Those likely to have attended a clinic were compared with 2015 actual LLINs allocation in the corresponding clinic to define, under-, over-, and matched-allocation. LLINs in over-allocated clinics were rationalized to the under-allocated clinics. Non-covered areas with a population of ≤100 were assigned to the nearest clinic within the existing distribution chain. Those with population >100, were assigned to a public clinic within the pre-determined *cut*-*off time* of the facilities not allocated LLINs in 2015.

## Results

**Fig. 3:**
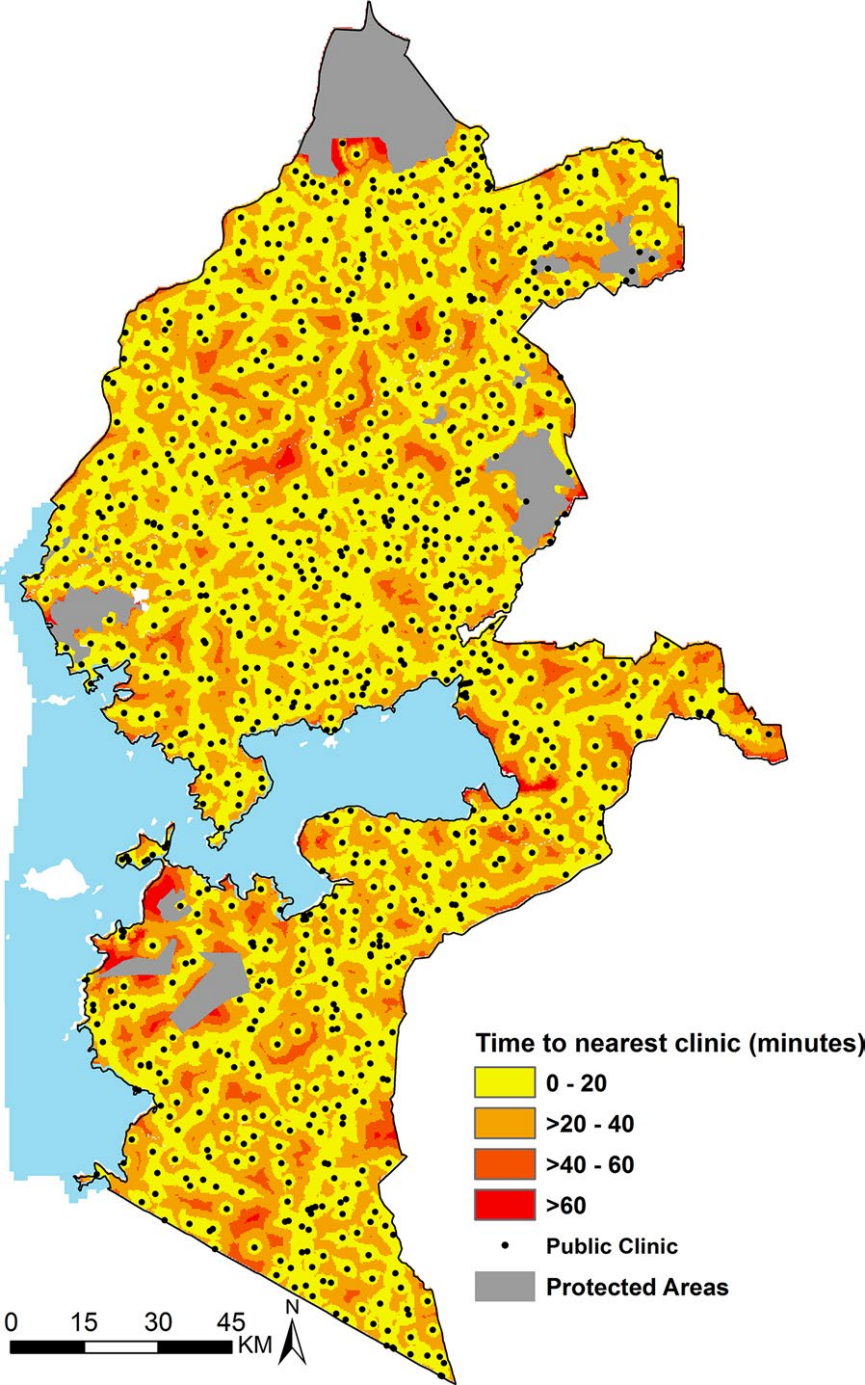
Map of Western Kenya showing travel time (in minutes) from each grid (300 × 300 m) to the nearest public health facility (*black dots*) distributing LLINs. Grouped travel time increases away from the facilities (*yellow* to *red*)

**Fig. 4:**
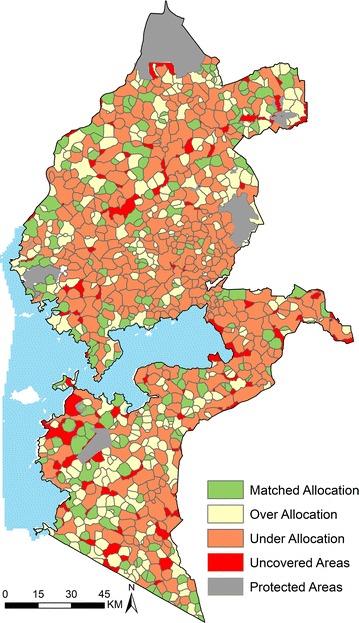
Map of Western Kenya showing distribution of 321 (36%) clinics over-allocated with 164,241 LLINs, 380 (43%) clinics with a deficiency of 255,628 LLINs and 187 (21%) clinics where allocation matched the need accounting for 86,990 LLINs. Areas outside the clinics catchment areas (non-covered areas) required approximately 43 clinics to have been allocated 17,703 LLINs to cater for the at risk population

The final list contained 1271 public health facilities out of which 888 had distributed LLINs in 2015. The analysis of the KDHS 2014 data showed that all 277 cluster points were within approximately 1-h of the nearest public clinic distributing LLINs (Fig. 3). ANC utilization decreased with increasing travel time, with attendance declining rapidly for distances greater than 40 min away from the nearest clinic on the decay curve. Catchment areas defined to include all those locations within 40 min of a health facility for the 888 public clinics distributing LLINs (exclusive of 13 clinics in islands of Lake Victoria) are shown in Fig. 4 and contained over 97% of pregnant women and infants in 2015 in the study area.

In the study counties, among women who had at least one live birth in the 5 years preceding the survey, over 97% had at least one ANC visit. As expected and exhaustively documented in literature within the Kenyan context [53–55], the final model of variables associated with ANC utilization showed that women of higher education and wealth quintile, those who were married, or had low parity or lived closer to a clinic were associated with higher odds of attending ANC (Table 2). All these variables varied spatially and this structure was used in interpolating them across the study area with 81–97% of pregnant women attending ANC clinics.

**Table 2:** Hierarchical mixed effects logistic regression model odds ratios of at least an ANC visit among women in the reproductive age (15–49 years) who had at a least a live birth, 5 years preceding the survey in Western Kenya in 2015 Kenya (N = 2776)

Variable	Description	OR (95% CI)
Fixed effect		
Time	Time to the nearest health facility	0.98 (0.95–1.00)
Household wealth	Poorest	Ref
	Poorer	1.53 (0.85–2.75)
	Middle	5.13 (2.12–12.44)
	Rich	8.43 (2.08–34.21)
	Richest	7.47 (0.81–68.67)
Maternal education	No education	Ref
	Primary	2.79 (0.87–8.90)
	Secondary and above	7.16 (1.70–30.17)
Parity	1 child	Ref
	2–3 children	0.35 (0.11–1.07)
	4–6 children	0.16 (0.05–0.51)
	7+ children	0.19 (0.05–0.68)
Marital status	Married or living with partner	Ref
	Divorced or separated or widowed	0.37 (0.18–0.77)
	Never in union	0.05 (0.02–0.14)

Random effect	Variance	Standard deviation
Cluster	1.1480	1.0714
Counties/DHS region	0.000005	0.0022

In 2015, 730,947 pregnant women and infants resided in the eight study counties with 97% (706,450) residing within modelled clinics catchment areas. The estimated LLINs need per clinic for 2015 was between 15 and 16,900, with 48% having a need of less than 500 while 23% with a need of more than 1000 LLINs annually. Among pregnant women and infants in catchment areas, 97% (682,377) were likely to have attended a clinic distributing LLINs representing 93% of the total population of pregnant women and infants in the study area.

**Fig. 5:**
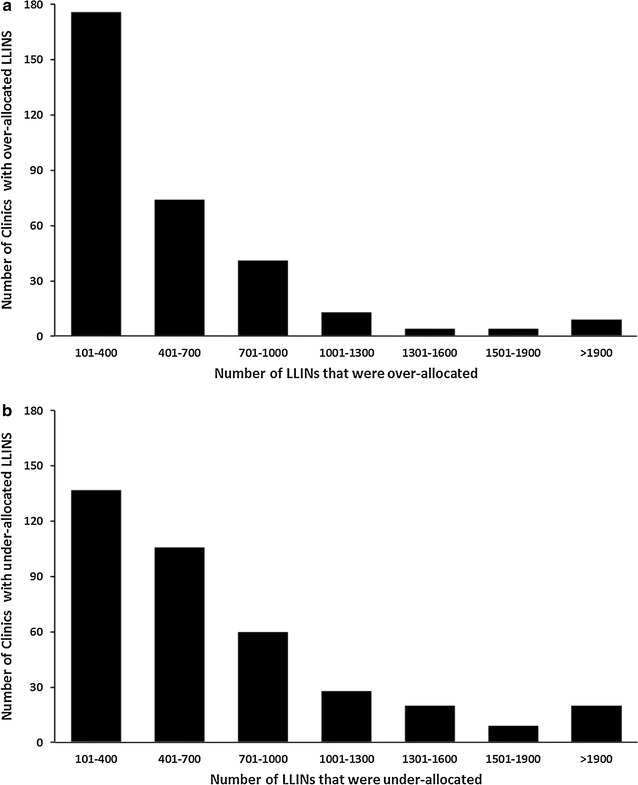
Number of clinics (*Y axis*) against number of LLINs that were over (**a**) and under-allocated (**b**) (*X axis*) in Western region of Kenya in 2015. For example, 176 clinics each had an over-allocation of between 101 and 400 LLINs while 60 clinics had an under-allocation ranging between 701 and 1000 LLINs

In 888 public health clinics, 591,880 LLINs were allocated in 2015. The number of LLINs allocated per clinic was between 10 and 6720. 53% of the clinics had less than 500 LLINs allocation per clinic while 19% had allocations greater than 1000 LLINs. Relative to an estimated overall need for 682,377 LLINs, the un-met need for 2015 was 90,497. The model suggested that 380 (43%) clinics were under-allocated by 255,628 LLINs (Fig. 4). The under-allocation per clinic was between 100 and >2000 LLINs (Fig. 5b). In 321 (36%) clinics (Fig. 4), 164,241 LLINs were over-allocated by up-to over 2000 LLINs (Fig. 5a) while 21% (187) of the clinics had a matched-allocation accounting for 86,990 LLINs (Fig. 4).

Using the spatial modelling framework to simulate an efficient LLIN allocation system, the 164, 241 over-allocated LLINs were redistributed reducing the shortfall from 255,628 to 91,387. Of the 95 areas that were outside 40 min and thus not covered by the LLIN distribution in 2015 (Fig. 4), 34 had a population of ≤100 and could be feasibly absorbed within the existing neighboring clinics resulting in increasing allocation of about 1606 LLINs to these clinics. 43 clinics not allocated LLINs in 2015, were within the determined 40 min *cut*-*off* time and would cater for 43 of remaining polygons equivalent to 17,703 extra LLINs. The remaining un-covered areas (18 polygons) all located between 40 and 60 min of the closest clinic distributing LLINs would require 5187 LLINs and accounts for less than 1% of those likely to attend a clinic.

## Discussion

A spatial modelling framework that can be adapted for the rational allocation of routinely distributed LLINs in Kenya was developed. LLIN need at each clinic was quantified by using a combination of modelled accessibility, population and ANC utilization and compared this to the actual allocations in 2015. Overall, the unmet need of LLINs for all pregnant women and infants and those likely to attend public clinics was 139,067 and 90,497 respectively. The current quantification approach of LLINs need covers approximately 88% of those likely to attend ANC clinics and about 81% of all pregnant women and infants in the study area. This shows that the routine distribution system in Western Kenya has a wide reach and is consistent with the Government of Kenya target of at least 80% of at risk population using appropriate malaria preventive intervention [56]. However, the facility level analysis also shows that there are important inequities in the distribution of LLINs to pregnant women and infants. The model provides a means for reducing such inequities and can be upgraded as consumption data improves.

Currently, the Ministry of Health relies on consumption data reported from health facilities and adjusted for estimated census projections of pregnant women and infants to quantify routine LLIN distribution. At delivery points, mechanisms for rational distribution have been rolled out including verification that a client qualifies for an LLIN, stamping the recipients’ booklet to avoid repeat collection from the same or another clinic and improved overall training of health workers on the management of commodities, regular auditing at the clinics and monthly reports. Recently, with the introduction of DHIS 2 [21, 57] in Kenya, LLINs consumption data are now reported through this system. This could potentially improve rational allocation, especially if predefined catchment areas and population maps such as those developed in this analysis are used as additional information to check whether distributions match the need for LLINs.

The definition of catchment areas could either be theoretical and based on simple proximity of the population to a health facility [58, 59] or could be improved with the available empirical data on utilization of services derived from household survey data rates [13, 41]. Previous studies have used only travel time and its relationship with reported utilization to define the probability of service use [13, 32]. However, in this study, other determinants of utilization such as wealth and maternal education, were used to capture both the spatial and social, demographic and economic factors that influence the use of ANC clinics. Arguably, this approach offers a more complete framework for quantifying the catchment population (likely ANC clients) with a broader catchment area [14].

Some important limitations are worth noting in the modelling framework used. First, the use of the nearest clinic when modelling travel times was assumed. This is because household survey data does not allow for the identification of the actual facility used by women and infants. It is likely that a proportion of these populations often use facilities that are not the closest for a variety of reasons, including the quality of services on offer [60]. In addition, all pregnant women were assumed to have an equal probability of getting an LLIN irrespective of the number of ANC visits (1+ ANC), however, pregnant women with 2 or more ANC visits may have an increased chance of receiving an LLIN in subsequent visits. Second, a threshold of 40 min was imposed in the definition of facility catchment areas but it is likely that this threshold is variable by facility and future models should account for them. Finally, there are uncertainties in the pregnancies and births density maps [61] used into compute the LLIN need. Live births were assumed to be are equivalent of infants, however, a neonatal mortality of 22 per 1000 live births in 2015 in Kenya [30] equivalent to 6429 deaths (2.2%) of all live births and 1.1% of all LLINs distributed in the study counties may empirically bias the estimation of need. However, the national policy planning makes allocation of live saving services on the basis that all avoidable deaths are averted and therefore services, such as LLINs, will not and should not be discounted on the basis on anticipated neonatal deaths. To ensure that all uncertainties are propagated within the modelling approach, Bayesian inference methods may provide a useful alternative, but these advantages must be balanced with the simplicity required in spatial modelling techniques in routine programme planning.

## Conclusion

The spatial modelling approach presented here offers a rational basis for spatial allocation of routine LLINs that is applicable to malaria endemic settings and, potentially, other maternal and child related commodities. The analysis shows that with a few minor adjustments in distribution and better rationalization, equity in LLIN distribution through the routine system could be achieved in Western Kenya where the threat of malaria is the greatest.

## Abbreviations

LLINs: long-lasting insecticidal nets
ITNs: insecticide-treated nets
ANC: antenatal care
DHIS 2: District Health Information System (version 2)
KDHS 2014: Kenya Demographic and Health Survey for the year 2014
DEM: digital elevation model
